# Comparative efficacy and safety of individual short-acting versus long-acting granulocyte colony-stimulating factors, including biosimilars, for primary prophylaxis of chemotherapy-induced myelosuppression in breast cancer patients: a systematic review and network meta-analysis protocol

**DOI:** 10.3389/fonc.2026.1764945

**Published:** 2026-03-24

**Authors:** Xiaoyun Bi, Shi Shu, Kangmin Tang, Ping Yin, Yuelai Chen

**Affiliations:** 1 Sleep Medicine Center, Longhua Hospital Shanghai University of Traditional Chinese Medicine, Shanghai, China; 2 School of Traditional Chinese Medicine, Shanghai University of Traditional Chinese Medicine, Shanghai, China; 3 School of Rehabilitation Science, Shanghai University of Traditional Chinese Medicine, Shanghai, China

**Keywords:** breast cancer, chemotherapy, granulocyte colony-stimulating factors, myelosuppression, network meta-analysis

## Abstract

**Introduction:**

Chemotherapy-induced myelosuppression (CIM), including neutropenia, thrombocytopenia, and anaemia, is a common and potentially life-threatening complication in breast cancer patients. Despite the clinical availability of both short-acting and long-acting Granulocyte Colony-Stimulating Factors (G-CSFs) for primary prophylaxis, evidence supporting the selection of the most appropriate therapeutic agent based on individual characteristics remains limited. This study employs a systematic review and network meta-analysis (NMA) to directly compare individualized G-CSF formulations, aiming to establish a definitive efficacy and safety ranking for clinical guidance.

**Methods and analysis:**

Randomised controlled trials of long-acting versus short-acting G-CSF therapy (both originator and biosimilar) in breast cancer patients with CIM will be identified via a search of PubMed, Embase, Web of Science, Cochrane Central Register for Controlled Trials, ClinicalTrials.gov, and the Chinese literature databases, CNKI, Wanfang, VIP, and SinoMed, from inception to 30 September 2025. The main outcomes include the myelosuppression incidence/severity (e.g., neutropenia, thrombocytopenia), chemotherapy relative dose intensity (or dose reductions/delays due to neutropenia), overall survival, Eastern Cooperative Oncology Group (ECOG) performance status, infection-related mortality, and adverse events. Two independent reviewers will perform document screening, study selection, and data extraction. The methodological quality of the included studies will be assessed using the Cochrane Risk of Bias tool (V.2.0). The certainty of the evidence will be rated using the Grading of Recommendations, Assessment, Development, and Evaluation System.

**Ethics and dissemination:**

This systematic review is based on already published data, so it does not require ethical approval. The findings of this review will be submitted to peer-reviewed journals.

## Background

Breast cancer, characterized by distinct epidemiological patterns and substantial heterogeneity, continues to be a leading cause of cancer-related mortality among women globally (1). Chemotherapy regimens with anthracyclines, cyclophosphamide, and paclitaxel have been the primary treatment for breast cancer patients for many years (2, 3). However, chemotherapy-induced myelosuppression (CIM) is one of the most prevalent dose-limiting toxicities in the treatment of breast cancer (3, 4). Severe myelosuppression can lead to complications like febrile neutropenia (FN), severe infections, anaemia due to impaired erythropoiesis, and thrombocytopenia-related bleeding risk. These complications can greatly raise the risk of hospitalization and often require lowering chemotherapy doses or delaying treatment. This could have an impact on the tumour’s treatment and the patients’ long-term survival (5, 6). Research indicates that one-third of breast cancer patients experience neutropenia following chemotherapy (7).

Granulocyte colony-stimulating factor (G-CSF) is a cytokine that is the primary regulator of granulocyte production and is produced by monocytes, macrophages, fibroblasts, endothelial cells, and bone marrow stromal cells. By encouraging the proliferation of bone marrow progenitor cells, G-CSF can enhance the production and maturation of granulocytes (8, 9). Based on pharmacokinetic properties, G-CSF can be categorized as short-acting formulations necessitating daily administration (e.g., filgrastim and lenograstim) and long-acting formulations requiring administration only once each treatment cycle (pegfilgrastim) (10). Clinical guidelines recommend the use of G-CSF for primary prophylaxis in patients receiving chemotherapy regimens with a high (>20%) risk of FN (7, 11–13).

Prior research has established a significant basis for the utilization of G-CSF in the prophylaxis of CIM. Many meta-analyses have investigated the effectiveness and primary preventative benefits of particular G-CSF formulations in individuals with breast cancer (14–16). But there are still certain challenges. For instance, as existing research predominantly focuses on specific drug combinations, the comparative effectiveness of different G-CSF regimens remains insufficiently evaluated (17). Moreover, short-acting and long-acting G-CSF formulations are grouped despite their heterogeneity, potentially obscuring differences between individual preparations (18). Furthermore, the inclusion of patients with diverse cancer diagnoses in certain studies limits the applicability of findings to specific patient subgroups, particularly those with breast cancer (19). Finally, outcome measures are primarily centred on FN, thereby failing to capture broader aspects of clinical management, such as chemotherapy relative dose intensity and the incidence or management of thrombocytopenia (20).

The introduction of new G-CSF formulations and the completion of additional clinical trials in recent years have expanded the database; however, the study results have demonstrated inconsistency (21). A randomized controlled trial (RCT) reported that differences in the incidence and duration of grade 3/4 neutropenia between the short-acting recombinant human G-CSF (rhG-CSF) and the long-acting polyethylene glycol-modified form (PEG-rhG-CSF) became increasingly evident across cycles 2–3 and reached statistical significance by cycle 4 (22). In contrast, another study observed no significant differences in the duration of grade 3/4 neutropenia or grade 4 neutropenia between the long-acting F-627 and short-acting filgrastim during cycles 2-4 (23). Likewise, direct comparisons of various G-CSFs reveal substantial heterogeneities in effectiveness and safety outcomes (24).

To address the existing evidence gap, this study introduces a systematic review and network meta-analysis (NMA) designed to directly compare individual G-CSF formulations. It will integrate direct and indirect comparison data to quantify relative effects and establish a clear efficacy ranking for these treatments in breast cancer patients undergoing primary prophylaxis of CIM. Focusing on this population and a comprehensive set of outcomes, the study aims to generate applicable evidence to improve clinical decision-making.

## Methods

### Study registration

Before data extraction, this protocol has been registered on PROSPERO under the number CRD420251153143, with a submission date of October 13, 2025. It is also developed in accordance with the guidelines established by the Preferred Reporting Items for Systematic Reviews and Meta-Analyses Protocol (PRISMA-P) declaration (25). The findings will be reported in conformance with the PRISMA extension for Network Meta-Analyses (PRISMA-NMA) checklist (26). The study is scheduled to begin on November 18, 2025, and end on October 8, 2026, for a period of one year.

### Eligibility criteria

The database will include only RCTs originally published in English or Chinese. Abstracts, editorials, clinical observations, case reports, cohort studies, non-randomized trials, case-control studies, and cross-sectional and retrospective studies will be omitted. Research on several cancer types will often be ignored unless it includes disaggregated data specific to breast cancer subgroups. To make sure that all relevant references are found and used, all available systematic reviews and meta-analyses will be looked at. **Table 1** shows the eligibility criteria, which include the study characteristics, population, intervention, comparator, and outcome measures.

**Table 1:** Eligibility criteria for included studies.

Category	Inclusion criteria	Exclusion criteria
Study characteristics	Comparing long-acting and short-acting G-CSF therapy; Published in English or Chinese; RCTs.	Abstracts, editorials, clinical observations, case studies, cohort studies, non-randomized trials, case-control studies, and investigations that do not qualify as experimental (including cross-sectional and retrospective studies) are also excluded.
Population	(a)Adult patients (≥18 years) with histologically confirmed breast cancer receiving G-CSF therapy for the primary prophylaxis of chemotherapy-induced myelosuppression. (b)There are no restrictions on race, age, national origin, education, or chemotherapy regimen.	Patients with concomitant other malignancies or bone marrow suppression not caused by chemotherapy.
Intervention	Prophylactic use of any short-acting or long-acting G-CSF, including originators and their biosimilars, as a single intervention in breast cancer patients receiving chemotherapy.	
Comparator	Placebo, standard care (no prophylactic G-CSF).	
Main outcomes	The main outcomes include the myelosuppression incidence/severity (e.g., neutropenia, thrombocytopenia), chemotherapy relative dose intensity (or dose reductions/delays due to neutropenia), overall survival, Eastern Cooperative Oncology Group (ECOG) performance status, infection-related mortality, and adverse events.	

### Data sources and search strategy

PubMed, Embase, Cochrane Central Register for Controlled Trials, Web of Science, ClinicalTrials.gov, and the Chinese literature databases are CNKI, Wanfang, VIP, and SinoMed, will be searched for articles from database inception to 30 September 2025. The search terms are focused on “Breast neoplasms”, “Breast cancer”, “Chemotherapy”, “Systemic therapy”, “Immunotherapy”, “Antibodies”, “Drug therapy”, “Myelosuppression”, “Bone Marrow Suppression”, “Neutropenia”, “Thrombocytopenia”, “Anaemia”, “Febrile Neutropenia”, “Granulocyte Colony-Stimulating Factor”, “G-CSF”, “Filgrastim”, “Pegfilgrastim”, “Lenograstim”, “MYL-1401H”, “Pegylated granulocyte colony-stimulating factor”, “Pegylated granulocyte colony-stimulating factor, human”, “Mecapegfilgrastim”, “Lipegfilgrastim”, “Neulasta”, “LA-EP2006”, “Zarxio”, “Neupogen”, “Tbo Filgrastim”, “Granix”, “Filgrastim-sndz”, “Ziextenzo”, “Fulphila”, “Udenyca”, “8MW0511”, “Eflapegrastim”, “Balugrastim”, “F-627”, “Telpegfilgrastim” and “Randomized Controlled Trial”. Searching for the subject words, subordinate words, and free words yields the final search formula. To find further research overlooked in the first search, the reference lists of all chosen papers will be separately examined. **Table 2** presents an example search strategy for the Cochrane Central Register for Controlled Trials. The search strategies for other databases are presented in the **Supplementary File**.

**Table 2:** Search strategy for the Cochrane central register for controlled trials.

Number	Search items
#1	MeSH descriptor: [Breast Neoplasms] explode all trees
#2	(Breast Neoplasm):ti,ab,kw OR (Neoplasm, Breast):ti,ab,kw OR (Neoplasms, Breast):ti,ab,kw OR (Breast Tumours):ti,ab,kw OR (Breast Tumour):ti,ab,kw OR (Tumour, Breast):ti,ab,kw OR (Tumours, Breast):ti,ab,kw OR (Breast Cancer):ti,ab,kw OR (Cancer, Breast):ti,ab,kw OR (Cancer of Breast):ti,ab,kw OR (Cancer of the Breast):ti,ab,kw OR (Malignant Neoplasm of Breast):ti,ab,kw OR (Breast Malignant Neoplasm):ti,ab,kw OR (Breast Malignant Neoplasms):ti,ab,kw OR (Malignant Tumour of Breast):ti,ab,kw OR (Breast Malignant Tumour):ti,ab,kw OR (Breast Malignant Tumours):ti,ab,kw OR (Mammary Cancer):ti,ab,kw OR (Cancer, Mammary):ti,ab,kw OR (Cancers, Mammary):ti,ab,kw OR (Mammary Cancers):ti,ab,kw OR (Mammary Neoplasms, Human):ti,ab,kw OR (Human Mammary Neoplasm):ti,ab,kw OR (Human Mammary Neoplasms):ti,ab,kw OR (Neoplasm, Human Mammary):ti,ab,kw OR (Neoplasms, Human Mammary):ti,ab,kw OR (Mammary Neoplasm, Human):ti,ab,kw OR (Breast Carcinoma):ti,ab,kw OR (Breast Carcinomas):ti,ab,kw OR (Carcinoma, Breast):ti,ab,kw OR (Carcinomas, Breast):ti,ab,kw OR (Mammary Carcinoma, Human):ti,ab,kw OR (Carcinoma, Human Mammary):ti,ab,kw OR (Carcinomas, Human Mammary):ti,ab,kw OR (human Mammary Carcinomas):ti,ab,kw OR (Mammary Carcinomas, Human):ti,ab,kw OR (Human Mammary Carcinoma):ti,ab,kw
#3	#1 OR #2
#4	MeSH descriptor: [Drug Therapy] explode all trees
#5	(Chemotherapies):ti,ab,kw OR (Pharmacotherapy):ti,ab,kw OR (Pharmacotherapies):ti,ab,kw OR (Therapy, Drug):ti,ab,kw OR (Drug Therapies):ti,ab,kw OR (Therapies, Drug):ti,ab,kw OR (Systemic therapy):ti,ab,kw OR (Immunotherapy):ti,ab,kw OR (Antibodies):ti,ab,kw
#6	MeSH descriptor: [Granulocyte Colony-Stimulating Factor] explode all trees
#7	(Factor, Granulocyte Colony-Stimulating):ti,ab,kw OR (Granulocyte Colony Stimulating Factor):ti,ab,kw OR (Colony-Stimulating Factor, Granulocyte):ti,ab,kw OR (Colony Stimulating Factor, Granulocyte):ti,ab,kw OR (Myeloid Growth Factor):ti,ab,kw OR (Factor, Myeloid Growth):ti,ab,kw OR (Growth Factor, Myeloid):ti,ab,kw OR (G-CSF):ti,ab,kw OR (Filgrastim):ti,ab,kw OR (Pegfilgrastim):ti,ab,kw OR (Lenograstim):ti,ab,kw OR (MYL-1401H):ti,ab,kw OR (Pegylated granulocyte colony-stimulating factor):ti,ab,kw OR (Pegylated granulocyte colony-stimulating factor, human):ti,ab,kw OR (Mecapegfilgrastim):ti,ab,kw OR (Lipegfilgrastim):ti,ab,kw OR (Efbemalenograstim alfa):ti,ab,kw OR (Neulasta):ti,ab,kw OR (LA-EP2006):ti,ab,kw OR (Zarxio):ti,ab,kw OR (Neupogen):ti,ab,kw OR (Tbo Filgrastim):ti,ab,kw OR (Granix):ti,ab,kw OR (Filgrastim-sndz):ti,ab,kw OR (Ziextenzo):ti,ab,kw OR (Fulphila):ti,ab,kw OR (Udenyca):ti,ab,kw OR (8MW0511):ti,ab,kw OR (Eflapegrastim):ti,ab,kw OR (Balugrastim):ti,ab,kw OR (F-627):ti,ab,kw OR (Telpegfilgrastim):ti,ab,kw
#8	(Bone Marrow Suppression):ti,ab,kw OR (Myelosuppression):ti,ab,kw OR (Neutropenia):ti,ab,kw OR (Neutropenias):ti,ab,kw OR (Thrombocytopenia):ti,ab,kw OR (Thrombocytopenias):ti,ab,kw OR (Thrombopenia):ti,ab,kw OR (Thrombopenias):ti,ab,kw OR OR (Anemia):ti,ab,kw OR (Anemias):ti,ab,kw OR (Febrile Neutropenia):ti,ab,kw OR (Febrile Neutropenias):ti,ab,kw OR (Neutropenia, Febrile):ti,ab,kw OR (Neutropenias, Febrile):ti,ab,kw
#9	#4 OR #5
#10	#6 OR #7
#11	#3 AND #8 AND #9 AND #10

### Selection of studies

**Figure 1:**
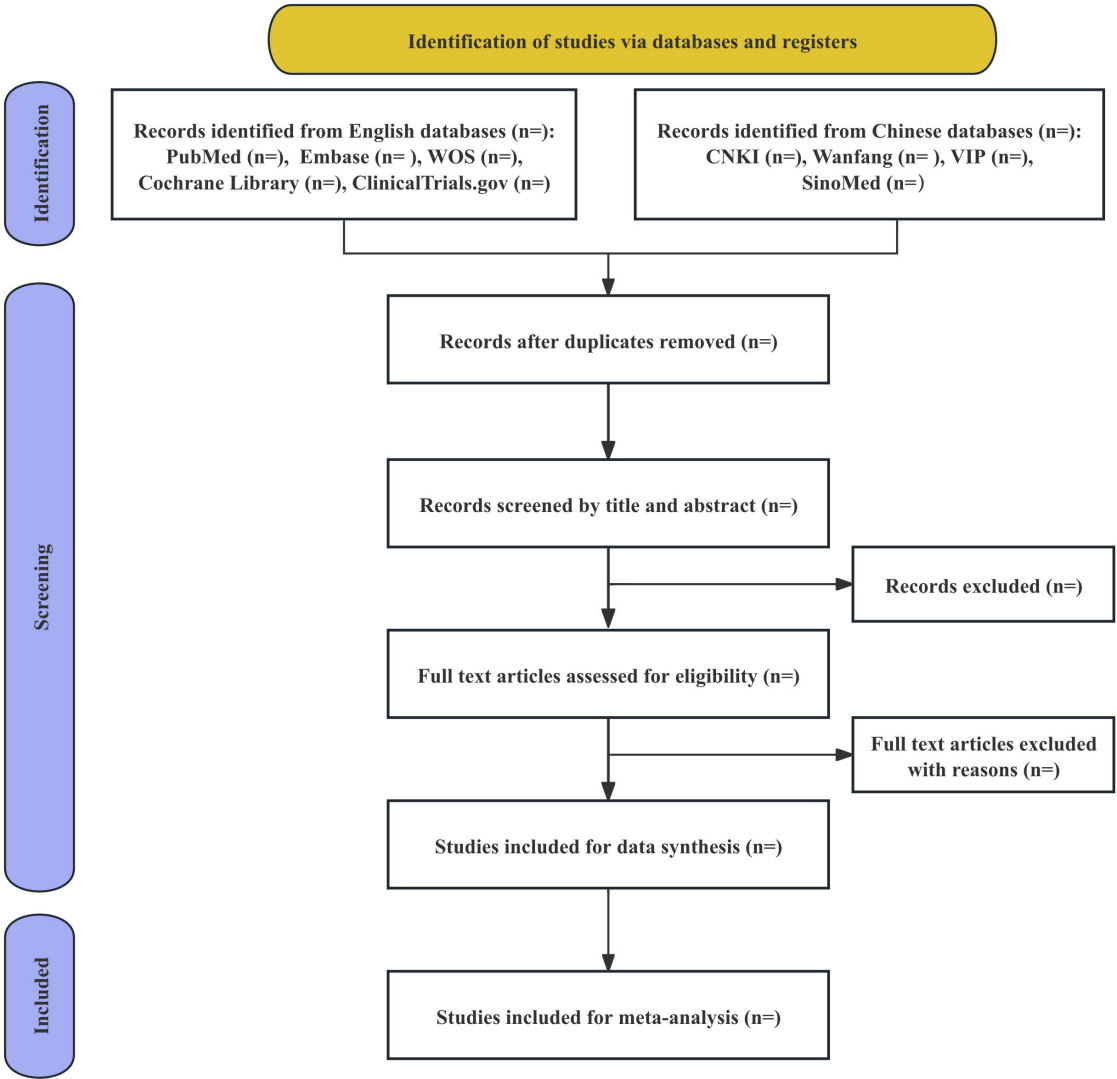
PRISMA flow diagram of the study selection process.

For initial eligibility, two researchers (XB and SS) will independently examine the abstracts and titles; a comprehensive full-text review will be used to determine which articles are ultimately qualified. The duplicates will be eliminated, and all retrieved records will be imported into EndNote V.21. A third reviewer (PY) will be consulted to settle any disputes amongst the researchers. **Figure 1** displays the selection procedure’s PRISMA flowchart.

### Data extraction and management

A structured data abstraction form will be utilized to enhance comprehension of the study characteristics, encompassing elements such as the title, first author, year of publication, and journal by XB and SS. Participant characteristics, including age, sample size, national origin, pathological type and stage, treatment history for breast cancer, and intervention measures (duration, frequency, and timing), will be documented. Outcomes concerning the safety and efficacy of the interventions will be extracted from the included studies. Pertinent outcome indicators and their associated measurement data will be incorporated, along with any accessible follow-up information. In cases of missing data or ambiguous information that could impact the analysis, the corresponding author will be approached to acquire comprehensive details. If it fails, missing data will be addressed systematically by utilizing accessible data sources, implementing suitable imputation techniques, and conducting sensitivity analyses (27). In the absence of variability measurements, the maximum standard deviation from comparable research utilizing the same measurement will be employed for the imputation approaches. If there is baseline and follow-up data but no mean change data, the correlation method will be used (28). The two researchers will verify the accuracy and consistency of the information retrieved upon its completion. All conflicts will be resolved through team discussions or by consulting the third researcher (PY).

### Risk of bias assessment

The risk of bias in the included studies will be independently assessed by two reviewers (XB and KT) using the Cochrane Risk of Bias (RoB) V.2.0 tool (https://www.cochrane.org/learn/courses-and-resources/cochrane-methodology/risk-bias) (29). The study’s quality will be assessed across five domains: (1) bias in the randomization process, (2) deviations from established interventions, (3) bias due to missing outcome data, (4) bias in outcome measurement, and (5) selective reporting of bias in results. Following each assessment, articles will be categorized as high risk, low risk, or of some concern.

### Evidence quality assessment

The Grading of Recommendations Assessment, Development and Evaluation (GRADE) framework will be employed to evaluate the validity of evidence for the main outcomes (30). The quality of the evidence will be categorized as “high,” “moderate,” “low,” and “very low” under the GRADE rating scale. Subsequently, the minimal contextualization framework of the GRADE approach will be employed to derive conclusions from the NMA. The framework delineates the quality of evidence in terms of imprecision, inconsistency, indirectness, publication bias, and study limitations (31).

### Statistical analysis

#### Pairwise meta-analysis

We will conduct a pairwise meta-analysis utilizing RevMan software V.5.4 (Review Manager, The Cochrane Collaboration, 2020) to compare the therapies with direct evidence. For dichotomous outcomes, the risk ratio (RR) with 95% confidence intervals (CIs) will be employed. We will use hazard ratios (HRs) with 95% CIs for time-to-event outcomes. Based on measurement scale consistency across studies, the mean difference (MD) or standardized mean difference (SMD) with 95% CIs will be calculated for continuous outcomes. Statistical heterogeneity among the studies in each pairwise meta-analysis will be quantified using the I² statistic and the τ² (tau-squared) parameter (32, 33). The I² statistic indicates the proportion of total variance among studies that may be ascribed to heterogeneity rather than random chance. The range is from 0% to 100%, with higher values indicating greater heterogeneity. The Cochrane Handbook states that I² values over 50% indicate substantial heterogeneity (34). The τ² parameter represents the estimated variance of true effect sizes across studies. Should a sufficient number of studies be available for a comparison, we will calculate a 95% prediction interval to illustrate the potential range of true effects in a new study (35).

#### Network meta-analysis

The Bayesian analysis will be performed using Markov Chain Monte Carlo (MCMC) methods, implemented with JAGS software (version 4.3.1) via the “rjags” and “gemtc” packages in R (version 4.5.1) (36, 37). Several distinct chains will be executed, each with a sufficiently large number of iterations to guarantee robust posterior sampling. The convergence of the model will be meticulously evaluated via visual analysis of trace plots and calculation of the Gelman-Rubin statistic (R̂), where values under 1.1 are deemed suggestive of satisfactory convergence (38, 39). In the network meta-analysis, treatment rankings will be summarized utilizing the Surface Under the Cumulative Ranking Curve (SUCRA), with elevated values signifying enhanced efficacy or superior performance (40). The node-splitting method will also be used to check how consistent direct and indirect evidence is for each relevant pairwise comparison. A p-value of less than 0.05 will be used to determine whether an inconsistency model is needed. If it is not, a consistency model will be kept (41).

#### Subgroup analysis and sensitivity analyses

To investigate possible causes of heterogeneity and evaluate the robustness of the results, subgroup analyses and sensitivity analyses will be performed. Subgroup analyses will particularly assess the impact of disease stage (early-stage vs. metastatic breast cancer), line of systemic therapy, treatment duration, follow-up duration, risk of myelosuppression associated with chemotherapeutic treatment, disease stage, and history of prior chemotherapy or radiotherapy. Additionally, should significant inconsistency be detected in the network meta-analysis, network meta-regression will be conducted utilizing these predetermined covariates to explore potential sources of heterogeneity and inconsistency (42).

### Publication bias assessment

A comparison-adjusted funnel plot (43) will be used to evaluate the presence of publication bias. Asymmetry in the plot or a statistically significant Egger’s test (44) (*P* < 0.05) will suggest the potential presence of publication bias or other small-study effects.

## Discussion

For patients with intermediate- and high-risk breast cancer requiring adjuvant or neoadjuvant chemotherapy, chemotherapy is a crucial component of their comprehensive treatment plan (45). G-CSF as a major preventative intervention is crucial for managing CIM, maintaining the desired chemotherapy dose intensity, and ensuring the efficacy of anti-tumour therapy. However, in actual practice, there is disagreement on whether short-acting or long-acting G-CSF is the better choice. Long-acting G-CSF has been commonly employed in recent years because of its convenience (one administration each cycle); however, short-acting G-CSF continues to be assessed for its adaptable administration schedule and possible cost-effectiveness.

This study aims to conduct a systematic review and NMA to integrate high-level evidence for the first time and directly compare the efficacy and safety of different individualized short-acting and long-acting G-CSF in the primary prophylaxis of CIM in breast cancer patients. By integrating direct and indirect comparison data, this study will quantify the relative effects of different G-CSF preparations and establish a clear efficacy ranking for all treatment measures.
