# Supplementary material for: Comparative efficacy and safety of individual short-acting versus long-acting granulocyte colony-stimulating factors, including biosimilars, for primary prophylaxis of chemotherapy-induced myelosuppression in breast cancer patients: a systematic review and network meta-analysis protocol
Source: Front Oncol. 2026 Mar 24;16:1764945. doi: 10.3389/fonc.2026.1764945 (PMC13054661; doi:10.3389/fonc.2026.1764945)
Supplement: Supplementary file 1 [file DataSheet1.doc]

**Supplemental file. Search strategies for each database**

**PubMed**

Search: (((((((((((((((((((((((((((((((((((((((((Breast Neoplasm[Title/Abstract]) OR (Neoplasm, Breast[Title/Abstract])) OR (Neoplasms, Breast[Title/Abstract])) OR (Breast Tumors[Title/Abstract])) OR (Breast Tumor[Title/Abstract])) OR (Tumor, Breast[Title/Abstract])) OR (Tumors, Breast[Title/Abstract])) OR (Breast Cancer[Title/Abstract])) OR (Cancer, Breast[Title/Abstract])) OR (Cancer of Breast[Title/Abstract])) OR (Cancer of the Breast[Title/Abstract])) OR (Malignant Neoplasm of Breast[Title/Abstract])) OR (Breast Malignant Neoplasm[Title/Abstract])) OR (Breast Malignant Neoplasms[Title/Abstract])) OR (Malignant Tumor of Breast[Title/Abstract])) OR (Breast Malignant Tumor[Title/Abstract])) OR (Breast Malignant Tumors[Title/Abstract])) OR (Mammary Cancer[Title/Abstract])) OR (Cancer, Mammary[Title/Abstract])) OR (Cancers, Mammary[Title/Abstract])) OR (Mammary Cancers[Title/Abstract])) OR (Mammary Neoplasms, Human[Title/Abstract])) OR (Human Mammary Neoplasm[Title/Abstract])) OR (Human Mammary Neoplasms[Title/Abstract])) OR (Neoplasm, Human Mammary[Title/Abstract])) OR (Neoplasms, Human Mammary[Title/Abstract])) OR (Mammary Neoplasm, Human[Title/Abstract])) OR (Breast Carcinoma[Title/Abstract])) OR (Breast Carcinomas[Title/Abstract])) OR (Carcinoma, Breast[Title/Abstract])) OR (Carcinomas, Breast[Title/Abstract])) OR (Mammary Carcinoma, Human[Title/Abstract])) OR (Carcinoma, Human Mammary[Title/Abstract])) OR (Carcinomas, Human Mammary[Title/Abstract])) OR (Human Mammary Carcinomas[Title/Abstract])) OR (Mammary Carcinomas, Human[Title/Abstract])) OR (Human Mammary Carcinoma[Title/Abstract])) OR ("Breast Neoplasms"[Mesh])) AND (((((((((((Chemotherapy[Title/Abstract]) OR (Chemotherapies[Title/Abstract])) OR (Pharmacotherapy[Title/Abstract])) OR (Pharmacotherapies[Title/Abstract])) OR ("Systemic therapy")) OR (Immunotherapy[Mesh])) OR (Antibodies[Mesh])) OR (Therapy, Drug[Title/Abstract])) OR (Drug Therapies[Title/Abstract])) OR (Therapies, Drug[Title/Abstract])) OR ("Drug Therapy"[Mesh]))) AND (("Granulocyte Colony-Stimulating Factor"[Mesh]) OR ((((((((((((((((((((((((((((((Factor, Granulocyte Colony-Stimulating[Title/Abstract]) OR (Granulocyte Colony Stimulating Factor[Title/Abstract])) OR (Colony-Stimulating Factor, Granulocyte[Title/Abstract])) OR (Colony Stimulating Factor, Granulocyte[Title/Abstract])) OR (Myeloid Growth Factor[Title/Abstract])) OR (Factor, Myeloid Growth[Title/Abstract])) OR (Growth Factor, Myeloid[Title/Abstract])) OR (G-CSF[Title/Abstract])) OR ("Filgrastim"[Mesh])) OR ("Pegfilgrastim" [Supplementary Concept])) OR ("Lenograstim"[Mesh])) OR ("MYL-1401H" [Supplementary Concept])) OR ("Pegylated granulocyte colony-stimulating factor" [Supplementary Concept])) OR ("Pegylated granulocyte colony-stimulating factor, human" [Supplementary Concept])) OR ("Mecapegfilgrastim")) OR ("Lipegfilgrastim")) OR ("Neulasta")) OR ("LA-EP2006")) OR ("Zarxio")) OR ("Neupogen")) OR ("Tbo Filgrastim")) OR ("Granix")) OR ("Filgrastim-sndz")) OR ("Ziextenzo")) OR ("Fulphila")) OR ("Udenyca")) OR ("8MW0511"[Title/Abstract])) OR ( "Eflapegrastim"[Title/Abstract])) OR ( "Balugrastim"[Title/Abstract])) OR ( "F-627"[Title/Abstract])) OR ("Telpegfilgrastim"[Title/Abstract])))) AND ((((((Bone Marrow Suppression) OR (Myelosuppression)) OR (("Neutropenia"[Mesh]) OR (Neutropenias))) OR (("Thrombocytopenia"[Mesh]) OR (((Thrombocytopenias[Title/Abstract]) OR (Thrombopenia[Title/Abstract])) OR (Thrombopenias[Title/Abstract])))) OR (("Anemia"[Mesh]) OR (Anemias))) OR (("Febrile Neutropenia"[Mesh]) OR (((Febrile Neutropenias[Title/Abstract]) OR (Neutropenia, Febrile[Title/Abstract])) OR (Neutropenias, Febrile[Title/Abstract]))))) AND (randomized controlled trial[Publication Type] OR randomized[Title/Abstract] OR placebo[Title/Abstract])

**Web of science**

#1: TS=("Breast Neoplasm" OR "Neoplasm, Breast" OR "Neoplasms, Breast" OR "Breast Tumor" OR "Breast Tumors" OR "Tumor, Breast" OR "Tumors, Breast" OR "Breast Cancer" OR "Cancer, Breast" OR "Cancer of Breast" OR "Cancer of the Breast" OR "Malignant Neoplasm of Breast" OR "Breast Malignant Neoplasm" OR "Breast Malignant Neoplasms" OR "Malignant Tumor of Breast" OR "Breast Malignant Tumor" OR "Breast Malignant Tumors" OR "Mammary Cancer" OR "Cancer, Mammary" OR "Cancers, Mammary" OR "Mammary Cancers" OR "Mammary Neoplasms, Human" OR "Human Mammary Neoplasm" OR "Human Mammary Neoplasms" OR "Neoplasm, Human Mammary" OR "Neoplasms, Human Mammary" OR "Mammary Neoplasm, Human" OR "Breast Carcinoma" OR "Breast Carcinomas" OR "Carcinoma, Breast" OR "Carcinomas, Breast" OR "Mammary Carcinoma, Human" OR "Carcinoma, Human Mammary" OR "Carcinomas, Human Mammary" OR "Human Mammary Carcinomas" OR "Mammary Carcinomas, Human" OR "Human Mammary Carcinoma")

#2: TS=("Chemotherapy" OR "Chemotherapies" OR "Pharmacotherapy" OR "Pharmacotherapies" OR "Therapy, Drug" OR "Drug Therapies" OR "Therapies, Drug" OR "Systemic therapy" OR "Immunotherapy" OR "Antibodies")

#3: TS=("Granulocyte Colony-Stimulating Factor" OR "Factor, Granulocyte Colony-Stimulating" OR "Granulocyte Colony Stimulating Factor" OR "Colony-Stimulating Factor, Granulocyte" OR "Colony Stimulating Factor, Granulocyte" OR "Myeloid Growth Factor" OR "Factor, Myeloid Growth" OR "Growth Factor, Myeloid" OR "G-CSF" OR "Filgrastim" OR "Pegfilgrastim" OR "Lenograstim" OR "MYL-1401H" OR "Pegylated granulocyte colony-stimulating factor" OR "Pegylated granulocyte colony-stimulating factor, human" OR "Mecapegfilgrastim" OR "Lipegfilgrastim" OR "Neulasta" OR "LA-EP2006" OR "Zarxio" OR "Neupogen" OR "Tbo Filgrastim" OR "Granix" OR "Filgrastim-sndz" OR "**Ziextenzo**" OR "**Fulphila**" OR "**Udenyca**" OR "8MW0511" OR "Eflapegrastim" OR "Balugrastim" OR "F-627" OR "Telpegfilgrastim")

#4: TS=("Bone Marrow Suppression" OR "Myelosuppression" OR "Neutropenia" OR "Neutropenias" OR "Thrombocytopenia" OR "Thrombocytopenias" OR "Thrombopenia" OR "Thrombopenias" OR "Anemia" OR "Anemias" OR "Febrile Neutropenia" OR "Febrile Neutropenias" OR "Neutropenia, Febrile" OR "Neutropenias, Febrile")

#5: TS=("randomized" OR "randomised" OR "randomization" OR "randomisation" OR "placebo" OR "randomized controlled trial" OR "RCT")

#6: #1 AND #2 AND #3 AND #4 AND #5

**EMBASE**

1. "breast tumor"/exp OR "breast tumor" OR "breast neoplasm":ab,ti OR "breast neoplasms":ab,ti OR "breast cancer":ab,ti OR "cancer of breast":ab,ti OR "cancer of the breast":ab,ti OR "malignant neoplasm of breast":ab,ti OR "breast malignant neoplasm":ab,ti OR "breast malignant neoplasms":ab,ti OR "malignant tumor of breast":ab,ti OR "breast malignant tumor":ab,ti OR "breast malignant tumors":ab,ti OR "mammary cancer":ab,ti OR "cancer, mammary":ab,ti OR "cancers, mammary":ab,ti OR "mammary cancers":ab,ti OR "mammary neoplasms, human":ab,ti OR "human mammary neoplasm":ab,ti OR "human mammary neoplasms":ab,ti OR "neoplasm, human mammary":ab,ti OR "neoplasms, human mammary":ab,ti OR "mammary neoplasm, human":ab,ti OR "breast carcinoma":ab,ti OR "breast carcinomas":ab,ti OR "carcinoma, breast":ab,ti OR "carcinomas, breast":ab,ti OR "mammary carcinoma, human":ab,ti OR "carcinoma, human mammary":ab,ti OR "carcinomas, human mammary":ab,ti OR "human mammary carcinomas":ab,ti OR "mammary carcinomas, human":ab,ti OR "human mammary carcinoma":ab,ti
2. "chemotherapy"/exp OR "chemotherapy" OR "chemotherapies":ab,ti OR "pharmacotherapy":ab,ti OR "pharmacotherapies":ab,ti OR "therapy, drug":ab,ti OR "drug therapies":ab,ti OR "therapies, drug":ab,ti OR "drug therapy"/exp OR "drug therapy":ab,ti OR "Systemic therapy":ab,ti OR "Immunotherapy":ab,ti OR "Antibodies":ab,ti
3. "granulocyte colony stimulating factor"/exp OR "granulocyte colony stimulating factor" OR "factor, granulocyte colony-stimulating":ab,ti OR "granulocyte colony stimulating factor":ab,ti OR "colony-stimulating factor, granulocyte":ab,ti OR "colony stimulating factor, granulocyte":ab,ti OR "myeloid growth factor":ab,ti OR "factor, myeloid growth":ab,ti OR "growth factor, myeloid":ab,ti OR "g-csf":ab,ti OR "Filgrastim":ab,ti OR "Pegfilgrastim":ab,ti OR "Lenograstim":ab,ti OR "MYL-1401H":ab,ti OR "Pegylated granulocyte colony-stimulating factor":ab,ti OR "Pegylated granulocyte colony-stimulating factor, human":ab,ti OR "Mecapegfilgrastim":ab,ti OR "Lipegfilgrastim":ab,ti OR "Neulasta":ab,ti OR "LA-EP2006":ab,ti OR "Zarxio":ab,ti OR "Neupogen":ab,ti OR "Tbo Filgrastim":ab,ti OR "Granix":ab,ti OR "Filgrastim-sndz":ab,ti OR "**Ziextenzo**":ab,ti OR "**Fulphila**":ab,ti OR "**Udenyca**":ab,ti OR "8MW0511":ab,ti OR "Eflapegrastim":ab,ti OR "Balugrastim":ab,ti OR "F-627":ab,ti OR "Telpegfilgrastim":ab,ti
4. "bone marrow suppression"/exp OR "bone marrow suppression" OR "myelosuppression":ab,ti OR "neutropenia"/exp OR "neutropenia":ab,ti OR "neutropenias":ab,ti OR "thrombocytopenia"/exp OR "thrombocytopenia":ab,ti OR "thrombocytopenias":ab,ti OR "thrombopenia":ab,ti OR "thrombopenias":ab,ti OR "anemia"/exp OR "anemia":ab,ti OR "anemias":ab,ti OR "febrile neutropenia"/exp OR "febrile neutropenia":ab,ti OR "febrile neutropenias":ab,ti OR "neutropenia, febrile":ab,ti OR "neutropenias, febrile":ab,ti
5. "randomized controlled trial"/exp OR "randomized controlled trial" OR "randomized":ab,ti OR "randomised":ab,ti OR "placebo":ab,ti
6. 1 and 2 and 3 and 4 and 5

**ClinicalTrials.gov**

((Breast Neoplasm) OR (Neoplasm, Breast) OR (Neoplasms, Breast) OR (Breast Tumors) OR (Breast Tumor) OR (Tumor, Breast) OR (Tumors, Breast) OR (Breast Cancer) OR (Cancer, Breast) OR (Cancer of Breast) OR (Cancer of the Breast) OR (Malignant Neoplasm of Breast) OR (Breast Malignant Neoplasm) OR (Breast Malignant Neoplasms) OR (Malignant Tumor of Breast) OR (Breast Malignant Tumor) OR (Breast Malignant Tumors) OR (Mammary Cancer) OR (Cancer, Mammary) OR (Cancers, Mammary) OR (Mammary Cancers) OR (Mammary Neoplasms, Human) OR (Human Mammary Neoplasm) OR (Human Mammary Neoplasms) OR (Neoplasm, Human Mammary) OR (Neoplasms, Human Mammary) OR (Mammary Neoplasm, Human) OR (Breast Carcinoma) OR (Breast Carcinomas) OR (Carcinoma, Breast) OR (Carcinomas, Breast) OR (Mammary Carcinoma, Human) OR (Carcinoma, Human Mammary) OR (Carcinomas, Human Mammary) OR (Human Mammary Carcinomas) OR (Mammary Carcinomas, Human) OR (Human Mammary Carcinoma)) AND ( (Chemotherapy) OR (Chemotherapies) OR (Pharmacotherapy) OR (Pharmacotherapies) OR (Therapy, Drug) OR (Drug Therapies) OR (Therapies, Drug) OR (Systemic therapy) OR (Immunotherapy) OR (Antibodies)) AND ((Granulocyte Colony-Stimulating Factor) OR (Factor, Granulocyte Colony-Stimulating) OR (Granulocyte Colony Stimulating Factor) OR (Colony-Stimulating Factor, Granulocyte) OR (Colony Stimulating Factor, Granulocyte) OR (Myeloid Growth Factor) OR (Factor, Myeloid Growth) OR (Growth Factor, Myeloid) OR (G-CSF) OR (Filgrastim) OR (Pegfilgrastim) OR (Lenograstim) OR (MYL-1401H) OR (Pegylated granulocyte colony-stimulating factor) OR (Pegylated granulocyte colony-stimulating factor, Human) OR (Mecapegfilgrastim) OR (Lipegfilgrastim) OR (Neulasta) OR (LA-EP2006) OR (Zarxio) OR (Neupogen) OR (Tbo Filgrastim) OR (Granix) OR (Filgrastim-sndz) OR (**Ziextenzo**) OR (**Fulphila**) OR (**Udenyca**) OR (8MW0511) OR (Eflapegrastim) OR (Balugrastim) OR (F-627) OR (Telpegfilgrastim)) AND ( (Bone Marrow Suppression) OR (Myelosuppression) OR (Neutropenia) OR (Neutropenias) OR (Thrombocytopenia) OR (Thrombocytopenias) OR (Thrombopenia) OR (Thrombopenias) OR (Anemia) OR (Anemias) OR (Febrile Neutropenia) OR (Febrile Neutropenias) OR (Neutropenia, Febrile) OR (Neutropenias, Febrile)) AND (randomized OR placebo)

**CNKI**

(TKA = "乳腺癌" OR "乳腺肿瘤" OR "乳腺恶性肿瘤" OR "乳癌" OR "乳岩") AND (TKA = "化疗" OR "化学治疗" OR "化学疗法" OR "化学药物治疗") AND (TKA = "粒细胞集落刺激因子" OR "G-CSF" OR "非格司亭" OR "重组人粒细胞集落刺激因子" OR "培非格司亭" OR "聚乙二醇非格司亭" OR "聚乙二醇化重组人粒细胞集落刺激因子" OR "来格司亭" OR "利培非格司亭" OR "硫培非格司亭" OR "美培格非司亭" OR "福富血" OR "优达佳" OR "优保津" OR "扎西奥" OR "替博非格司亭" OR "8MW0511" OR "Eflapegrastim" OR "Balugrastim" OR "F-627" OR "Telpegfilgrastim" ) AND (TKA = "骨髓抑制" OR "中性粒细胞减少" OR "粒细胞减少" OR "发热性中性粒细胞减少" OR "白细胞减少" OR "血小板减少" OR "贫血") AND (TKA ="随机" OR "随机对照" OR "RCT" OR "随机对照试验")

**Wanfang**

(主题:("乳腺癌" OR "乳腺肿瘤" OR "乳腺恶性肿瘤" OR "乳癌" OR "乳岩")) AND (主题:("化疗" OR "化学治疗" OR "化学疗法" OR "化学药物治疗")) AND (主题:("粒细胞集落刺激因子" OR "G-CSF" OR "非格司亭" OR "重组人粒细胞集落刺激因子" OR "培非格司亭" OR "聚乙二醇非格司亭" OR "聚乙二醇化重组人粒细胞集落刺激因子" OR "来格司亭" OR "利培非格司亭" OR "硫培非格司亭" OR "美培格非司亭" OR "福富血" OR "优达佳" OR "优保津" OR "扎西奥" OR "替博非格司亭" OR "8MW0511" OR "Eflapegrastim" OR "Balugrastim" OR "F-627" OR "Telpegfilgrastim" )) AND (主题:("骨髓抑制" OR "中性粒细胞减少" OR "粒细胞减少" OR "发热性中性粒细胞减少" OR "白细胞减少" OR "血小板减少" OR "贫血")) AND (主题:("随机" OR "随机对照" OR "RCT" OR "随机对照试验"))

**VIP**

((((((((((摘要=乳腺癌 OR 摘要=breast cancer) OR 摘要=breast carcinoma) OR 摘要=mammary cancer) OR 摘要=乳岩) OR 摘要=乳癌) OR 摘要=乳腺肿瘤) AND ((((((((摘要=化疗 OR 摘要=chemical therapy) OR 摘要=chemo therapy) OR 摘要=chemotherapeutic) OR 摘要=chemotherapy) OR 摘要=chemotherapy combined) OR 摘要=化学疗法) OR 摘要=化学药物治疗) OR 摘要=化学治疗)) AND (摘要=粒细胞集落刺激因子 OR 摘要=G-CSF OR 摘要=非格司亭 OR 摘要=重组人粒细胞集落刺激因子 OR 摘要=培非格司亭 OR 摘要=聚乙二醇非格司亭 OR 摘要=聚乙二醇化重组人粒细胞集落刺激因子 OR 摘要=来格司亭 OR 摘要=利培非格司亭 OR 摘要=硫培非格司亭 OR 摘要=美培格非司亭 OR 摘要=福富血 OR 摘要=优达佳 OR 摘要=优保津 OR 摘要=扎西奥 OR 摘要=替博非格司亭 OR 摘要=8MW0511 OR 摘要=Eflapegrastim OR 摘要=Balugrastim OR 摘要=F-627 OR 摘要=Telpegfilgrastim)) AND (=NULL ((((((摘要=骨髓抑制 OR 摘要=中性粒细胞减少) OR 摘要=粒细胞减少) OR 摘要=发热性中性粒细胞减少) OR 摘要=白细胞减少) OR 摘要=贫血) OR 摘要=血小板减少))) AND ((((摘要=随机对照 OR 摘要=随机分组) OR 摘要=随机) OR 摘要=rct) OR 摘要=RCT))

**SinoMed**

("乳腺癌"[常用字段] OR "乳腺肿瘤"[常用字段] OR "Breast Neoplasms"[常用字段] OR "乳腺瘤"[常用字段] OR "乳腺癌症"[常用字段] OR "人类乳腺肿瘤"[常用字段] OR "乳腺肿瘤"[主题词]) AND "化疗"[常用字段] AND ("白细胞减少"[常用字段] OR "Leukopenia"[常用字段] OR "白血球减少"[常用字段] OR "白细胞减少"[主题词] OR "骨髓抑制"[常用字段] OR "中性粒细胞减少"[常用字段] OR "Neutropenia"[常用字段] OR "中性粒细胞减少"[主题词] OR "贫血"[常用字段] OR "Anemia"[常用字段] OR "贫血"[主题词] OR "血小板减少"[常用字段] OR "Thrombocytopenia"[常用字段] OR "血小板减少症"[常用字段] OR "血小板减少"[主题词]) AND "随机"[常用字段] AND ("粒细胞集落刺激因子"[常用字段] OR "Granulocyte Colony-Stimulating Factor"[常用字段] OR "G-CSF"[常用字段] OR "粒细胞集落刺激因子"[主题词] OR "非格司亭"[常用字段] OR "重组人粒细胞集落刺激因子"[常用字段] OR "培非格司亭"[常用字段] OR "聚乙二醇非格司亭"[常用字段] OR "聚乙二醇化重组人粒细胞集落刺激因子"[常用字段] OR "来格司亭"[常用字段] OR "利培非格司亭"[常用字段] OR "硫培非格司亭"[常用字段] OR "美培格非司亭"[常用字段] OR "福富血"[常用字段] OR "优达佳"[常用字段] OR "优保津"[常用字段] OR "扎西奥"[常用字段] OR "替博非格司亭"[常用字段] OR "8MW0511"[常用字段] OR "Eflapegrastim"[常用字段] OR "Balugrastim"[常用字段] OR "F-627"[常用字段] OR "Telpegfilgrastim"[常用字段] )
